# Design of Fractional Order Odd-Harmonics Repetitive Controller for Discrete-Time Linear Systems with Experimental Validations

**DOI:** 10.3390/s22228873

**Published:** 2022-11-16

**Authors:** Edi Kurniawan, Jalu A. Prakosa, Hai Wang, Sensus Wijonarko, Tatik Maftukhah, Purwowibowo Purwowibowo, Harry Septanto, Enggar B. Pratiwi, Dadang Rustandi

**Affiliations:** 1 Research Center for Photonics, National Research and Innovation Agency, Tangerang Selatan 15314, Indonesia; 2 Discipline of Engineering and Energy, Murdoch University, Perth, WA 6150, Australia; 3 Research Center for Smart Mechatronics, National Research and Innovation Agency, Bandung 40135, Indonesia

**Keywords:** repetitive controller, odd-harmonics internal model, fractional stabilizing controller, optimization

## Abstract

This paper presents a simple and straightforward design of a discrete-time fractional-order odd-harmonics repetitive controller (RC). Unlike general RC designs, the proposed method utilizes an internal model with a half-period delay and a stabilizing controller with a fractional phase lead compensator. First, the odd-harmonics internal model representing odd-harmonics frequencies is constructed by using the information of the reference’s basis period and the preferred tracking bandwidth. Secondly, an optimization problem synthesized from the stability condition of the RC closed-loop system is solved to obtain the fractional phase lead compensator. Finally, the fractional term of the stabilizing controller is realized by using a causal and stable infinite impulse response (IIR) filter, where the filter coefficients are computed by applying the Thiran formula. Simulation and experimental validation on a servomotor system are conducted to verify the effectiveness of the proposed design.

## 1. Introduction

Repetitive controller (RC) as a well-known control strategy was first developed by Inoue et al. [1] for high accuracy control of a power supply. RC is a learning-type controller like iterative learning controller used for tracking control or rejection of periodic signals, which has been developed for many control applications such as precision irrigation [2,3], mechatronics [4,5], renewable energy [6,7], power electronics [8,9] and biomedical [10,11]. The superior performance of RC is due to the use of an internal model as proposed by Francis and Wohnam [12]. The internal model represents the periodic reference/disturbance model and behaves as a periodic signal generator for tracking reference/compensating disturbance signal with a zero-tracking error.

A general discrete-time internal model is constructed by a one-cycle delay $z^{-N}$ with positive feedback forming the transfer function of $z^{-N}/1-z^{-N}$. Note that *N* is generally an integer number representing the number of time steps per reference/disturbance period. Then, a low-pass filter is usually cascaded to $z^{-N}$ for improving the robustness of the RC system, but at the expense of tracking accuracies at higher frequencies. This type of internal model offers a zero steady-state error for tracking/rejecting periodic signals at fundamental frequency, even-and-odd harmonics components. In many real situations, reference/disturbance signals generally involve odd-harmonics only. These situations can be found in applications such as power electronic systems [8,9,13,14], magnetic rotor systems [15,16], nano-positioning systems [17], field-modulated magnetometer systems [18] and centrifugal compressors [19]. Inspired by this fact, the use of a general internal model is unnecessary if we only target odd-harmonics periodic components. If the general internal model is applied in these situations, then infinite gains will be introduced at even harmonics components too, which can reduce the system robustness and degrade the system performance [20]. In addition, the general internal model also introduces slow transient responses due to the presence of a one-cycle delay term $z^{-N}$.

To deal with these situations, an odd-harmonics internal model was proposed in [20], aiming to provide infinite gains only at odd-harmonic frequencies. This implies that the infinite gains are introduced at the targeted odd-harmonic frequencies. Unlike the general internal model, the odd-harmonics uses a half-period delay $z^{-N/2}$, which improves the transient performance of the RC system. Besides the internal model, the stabilizing controller is also required to construct the RC system. The stabilizing controller is required to guarantee the stability of the closed-loop RC system. Moreover, the stabilizing controller also determines the convergence rate of the system error. The stabilizing controller is sometimes designed as the inverse of the plant model/closed-plant model [21,22,23], which is often not available due to the plant uncertainties and disturbances [24]. In addition, the complexity of the stabilizing controller is easily increased when the plant has a higher-order model. A pole placement-based design method was presented in [25,26], where the numerator and denominator of the controller are acquired by solving the Diophantine Equation. This design method yields a stabilizing controller with an order as high as the internal model [27]. In [24,28], the stabilizing controller was designed as a phase lead compensator $K_{p}z^{m}$, where $K_{p}$ is the learning gain and *m* is a lead-step integer. The design task involves examining various phase responses for every lead step *m* trial in order to determine the order *m* that provides a larger stable bandwidth. Moreover, the use of an integer lead step *m* results in non-flexible phase compensation.

In this work, we develop a new and novel approach to designing the stabilizing controller for the discrete-time odd-harmonics RC system. An optimization-based design methodology is employed to obtain the stabilizing controller in the simple form of a phase lead compensator. Unlike the conventional phase lead compensator in [24,28], we use a fractional order phase lead compensator, where the compensator’s parameters are chosen by solving the optimization problem. Hence, this design method is straightforward because it avoids the manual tuning process in the frequency domain to obtain the stabilizing controller as shown in [24,28]. A fractional order stabilizing controller is considered here in order to provide more flexible phase compensation. Here, the fractional part is implemented by using a causal and stable IIR filter, whose filter coefficients are computed by applying the Thiran formula. Simulation and experimental results are demonstrated to verify the superior performance of the proposed design. In order to highlight the originality of our research work, the contributions to this work are listed as follows:

1. An internal model with half-cycle delay representing odd-harmonic periodic signals is used to provide faster transient response.
2. An optimization-based design methodology is developed to obtain the fractional order stabilizing controller.
3. The fractional order stabilizing controller is realizable since the fractional part of the controller is approximated by using a stable and causal IIR filter.

The remainder of this paper is structured as follows. Section 2 explains the problem statement and preliminaries underlying a repetitive control problem and a general design of repetitive controller. In Section 3, the proposed design is described, covering the structure, stability analysis, synthesis and realization of the fractional-order odd-harmonics repetitive controller. Section 4 presents the simulation results, followed by experimental validation in Section 5. A comparison study is also discussed in Section 5. Lastly, the conclusion is drawn in Section 6.

## 2. Problem Statement and Preliminaries

### 2.1. Repetitive Control Problem

In this work, we consider a discrete-time linear time invariant (LTI) system represented as follows:

(1) $$\begin{array}{c} Y\left(z\right)=\left[P(z)\right]U\left(z\right)+V\left(z\right), \end{array}$$

where U(z), Y(z) and V(z) are the Z-transforms of discrete-time signals of u(k), y(k) and v(k), respectively, and P(z) is the plant model. Here, u(k), y(k), v(k)∈R, respectively, denote a control input, a plant output and an input disturbance.

Suppose that the LTI system (1) is required to track a periodic reference r(k) or/and reject a repetitive disturbance v(k) such that the tracking error e(k) converges to zero. The tracking error e(k) is defined as

(2) $$\begin{array}{c} e(k):=r(k)-y(k). \end{array}$$

The design objective is to synthesize the control input u(k) such that the reference r(k) is tracked, the disturbance v(k) is cancelled, the tracking error e(k) quickly converges to zero steady-state, and the resulting closed-loop system is both stable and has faster transient response. Note that r(k) and v(k) are periodic signals having odd-harmonics components with a similar fundamental frequency.

### 2.2. General Repetitive Controller

**Figure 1:**
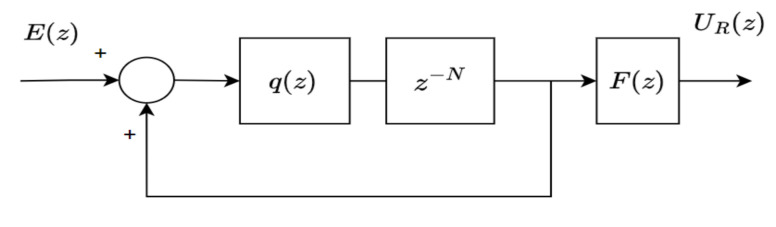
A general repetitive controller.

Suppose that the system (1) is subject to the periodic reference r(k) and/or repetitive disturbance v(k) with a basis frequency of $f_{b}=f_{r}=f_{v}$. Note that $f_{r}$ and $f_{b}$ are the fundamental frequencies of reference and disturbance, respectively. The repetitive controller depicted in Figure 1 can be used to form a stable closed-loop system offering an accurate reference tracking and/or a good disturbance rejection.

The controller shown in Figure 1 is then referred to as general RC, which is equivalent to the following transfer function:

(3) $$\begin{array}{c} C_{g}\left(z\right)=\frac{U_{R}\left(z\right)}{E(z)}=\left(\frac{q\left(z\right)z^{-N}}{1-q\left(z\right)z^{-N}}\right)F\left(z\right), \end{array}$$

where E(z) is an error signal, $U_{R}\left(z\right)$ is a repetitive control signal, q(z) is a low-pass filter, F(z) is a stabilizing controller and $N=\frac{T_{b}}{T_{s}}$ is an integer number of samples per reference period with $T_{s}$ being the sampling period and $T_{b}$ being the reference period. The term $\left[q\left(z\right)z^{-N}/\left(1-q\left(z\right)z^{-N}\right)\right]$ in (3) represents the general RC’s internal model, defined as

(4) $$\begin{array}{c} I_{g}\left(z\right)=\frac{q\left(z\right)z^{-N}}{1-q\left(z\right)z^{-N}}. \end{array}$$

The internal model (4) has *N* evenly spaced poles on the unit circle at the harmonics of the basis frequency $f_{b}$. The filter q(z) in (4) aims to improve the system robustness against unmodeled dynamics at the high frequencies. The presence of q(z) pushes the poles at the higher frequencies toward inside of the unit circle. Since the poles of (4) are mostly located at the unit circle, the internal model (4) has a capacity to perfectly track/compensate any periodic signal with frequency of $nf_{b}<f_{q}$, where $n=1,2,\cdots ,n_{m}$, $f_{q}$ is the q(z)’s bandwidth and $n_{m}f_{b}<f_{q}$ [27].

## 3. Proposed Method

### 3.1. Controller Structure

**Figure 2:**
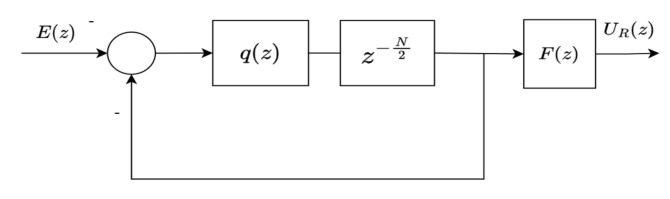
An odd-harmonics repetitive controller.

An odd-harmonics repetitive controller (OHRC) has a controller structure as shown in Figure 2. Unlike general RC in Figure 1, the OHRC has negative input and negative feedback. In addition, the OHRC utilizes a half-cycle delay $z^{-N/2}$.

The input–output relation of the OHRC is then given as

(5) $$\begin{array}{c} C_{o}\left(z\right)=\underset{\mathrm{internal}\mathrm{model}}{\underset{︸}{\frac{-q\left(z\right)z^{-N/2}}{1+q\left(z\right)z^{-N/2}}}}\;F\left(z\right), \end{array}$$

where $C_{o}\left(z\right)$ is defined as the transfer function of OHRC, q(z) is q-filter and F(z) is a stabilizing controller.

**Figure 3:**
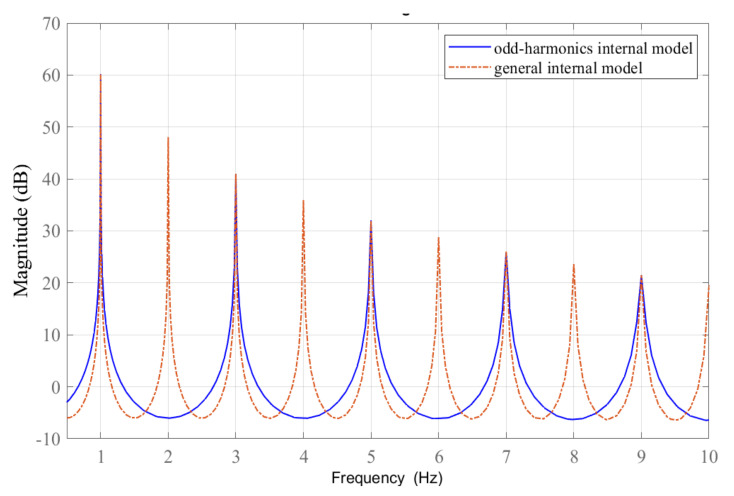
Magnitude responses of the odd-harmonics and general internal models with a basis frequency of 1 Hz (2π rad/s).

Similar to (3), the filter q(z) is inserted to improve the robustness of the OHRC system. This is due to the pure internal model such as $\left[-z^{-N/2}/\left(1+z^{-N/2}\right)\right]$ being also susceptible to the instabilities. Here, the filter q(z) is chosen as moving average filter as follows:

(6) $$\begin{array}{c} q\left(z\right)=\sum_{l=0}^{h}q_{l}z^{l}+\sum_{l=1}^{h}q_{l}z^{-l} \end{array}$$

where $0<q_{l}<1$ and $\sum_{l=1}^{h}q_{l}+q_{0}=1$. The filter q(z) in (6) is a low-pass filter contributing zero-phase for all frequency components and unity gain at the frequencies below the filter bandwidth. Note that the term $\left[\frac{-q\left(z\right)z^{-N/2}}{1+q\left(z\right)z^{-N/2}}\right]$ in (5) corresponds to an internal model design proposed in [20]. This internal model represents the reference models at the odd-harmonics frequencies only, i.e., $f\in \left[f_{b},3f_{b},\cdots ,\left(N/2-1\right)f_{b}\right]$. This behavior can be seen from Figure 3, indicating the magnitude responses of odd-harmonics and general internal models with a basis frequency of 1 Hz. Observing the infinite gains of OHRC, it is obvious that the trajectories at the desired odd-harmonics only will be tracked.

In the RC-controlled system, the stabilizing controller F(z) is compulsory to ensure the stability of the RC closed-loop system. In addition, the stabilizing controller also determines the convergence rate of the system error. A novel and new design of stabilizing controller F(z) is covered in Section 3.3, i.e., Fractional Order Stabilizing Controller.

### 3.2. Stability Analysis

**Figure 4:**
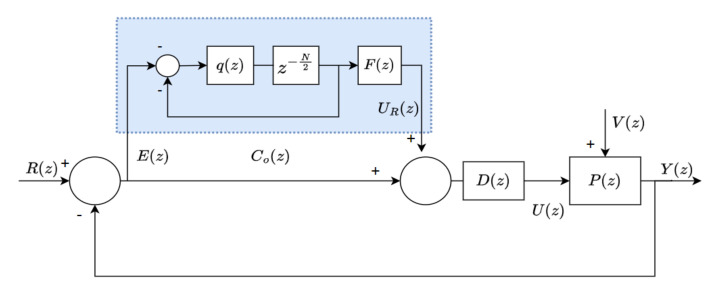
Plug-in OHRC system.

In this subsection, the stability conditions of the plug-in OHRC system are analyzed. The stability conditions are then used to design our proposed stabilizing controller. The block diagram of the overall control system is shown in Figure 4, where P(z) is the plant model, D(z) is the feedback controller, $C_{o}\left(z\right)$ is the OHRC, R(z),U(z),V(z),Y(z)∈R are, respectively, the reference, control input, disturbance and output signal.

The sensitivity function of the closed-loop OHRC system shown in Figure 4 is

(7) $$\begin{array}{c} \frac{E(z)}{R(z)}=\frac{1}{1+\{1+C_{o}\left(z\right)\}D\left(z\right)P\left(z\right)} \end{array}$$

Substituting (5) into (7), we have

(8) $$\begin{array}{c} \frac{E(z)}{R(z)}=\frac{1+q\left(z\right)z^{-N/2}}{1+q\left(z\right)z^{-N/2}+\left\{1+q\left(z\right)z^{-N/2}-F\left(z\right)q\left(z\right)z^{-N/2}\right\}D\left(z\right)P\left(z\right)} \end{array}$$

Simplifying the denominator part of (8), we get

(9) $$\begin{array}{c} \frac{E(z)}{R(z)}=\frac{1+q\left(z\right)z^{-N/2}}{\left[1+D(z)P(z)\right]\left[1+\left\{1-F\left(z\right)\frac{D(z)P(z)}{1+D(z)P(z)}\right\}q\left(z\right)z^{-N/2}\right]} \end{array}$$

Let us define the stabilized plant model $P_{s}\left(z\right)$ given by

(10) $$\begin{array}{c} P_{s}\left(z\right)=\frac{D(z)P(z)}{1+D(z)P(z)}. \end{array}$$

Then, (9) can be rewritten as

(11) $$\begin{array}{c} \frac{E(z)}{R(z)}=\frac{\left[1+q\left(z\right)z^{-N/2}\right]}{\left[1+D(z)P(z)\right]\left[1+\left\{1-F\left(z\right)P_{s}\left(z\right)\right\}q\left(z\right)z^{-N/2}\right]}, \end{array}$$

which can be factorized into three parts as follows:

(12) $$\begin{array}{c} \frac{E(z)}{R(z)}=\underset{\mathrm{Part}A}{\underset{︸}{\left[1+q\left(z\right)z^{-N/2}\right]}}\;\underset{\mathrm{Part}B}{\underset{︸}{\left[\frac{1}{1+D(z)P(z)}\right]}}\;\underset{\mathrm{Part}C}{\underset{︸}{\left[\frac{1}{1+\left\{1-F\left(z\right)P_{s}\left(z\right)\right\}q\left(z\right)z^{-N/2}}\right]}}\;. \end{array}$$

Based on (9), the plug-in OHRC system in Figure 4 is internally stable if the following conditions are satisfied [20]:

– (**C1**): q(z) is stable.
– (**C2**): $1/\left[1+D(z)P(z)\right]$ is stable.
– (**C3**): ${∥\{1-F\left(z\right)P_{s}\left(z\right)\}q\left(z\right)∥}_{\infty }<1$, which also can be expressed as
  (13) $$\begin{array}{c} {∥\{1-F\left(z\right)P_{s}\left(z\right)\}q\left(z\right)∥}_{\infty }\leq {∥1-F\left(z\right)P_{s}\left(z\right)∥}_{\infty }{∥q(z)∥}_{\infty }<1. \end{array}$$

Note that the notation ${∥X(z)∥}_{\infty }$ represents the $H_{\infty }$-norm of the transfer function X(z), which can be interpreted as the maximum value of the magnitude responses of X(z) for all frequencies (i.e., $\forall \;z=e^{j\omega }\;,0<\omega <\pi /T$).

**Remark 1.**  ***C1*** *and* ***C2*** *are, respectively, needed to ensure the stability of Parts A and B in (12). In addition,* ***C2*** *also implies that the closed-loop plant model $P_{s}\left(z\right)$ in (10) is a stable transfer function.* ***C3*** *is derived by using small gain theorem assuring the stability of Part C (12).*

### 3.3. Fractional Order Stabilizing Controller

Several assumptions are made before proceeding to the design of stabilizing controller F(z):

**Assumption 1.**  *The filter q(z) is chosen as a stable low-pass filter giving the unity gain at the referred bandwidth $\omega_{q}$ (i.e., $N_{q}\left(\omega \right)=1\;\mathrm{for}\;\mathrm{all}\;\omega \;\mathrm{that}\;\mathrm{satisfy}\;0<\omega <\omega_{q}$ and $N_{q}\left(\omega \right)\ll 1\;\mathrm{for}\;\mathrm{all}\;\omega \;\mathrm{that}\;\mathrm{satisfy}\;\omega_{q}<\omega <\pi /T$, where $\omega_{q}=2\pi f_{q}$). In addition, q(z) contributes zero-phase for all frequency components (i.e., $\theta_{q}\left(\omega \right)=0^{o}\;\mathrm{for}\;\mathrm{all}\;\omega \;\mathrm{that}\;\mathrm{satisy}\;0<\omega <\pi /T$). Here, $N_{q}\left(\omega \right)$ and $\theta_{q}\left(\omega \right)$, respectively, represent the magnitude and phase characteristics of q(z).*

**Assumption 2.**  *The plant model P(z) is known and the feedback controller D(z) is chosen such that the $P_{s}\left(z\right)$ in (10) has a stable transfer function. This implies that $1/\left[1+D(z)P(z)\right]$ is also stable.*

**Assumption 3.**  *The reference R(z) and disturbance V(z) are periodic with a common basis frequency $\omega_{b}$, i.e., $\omega_{b}=2\pi /T_{b}$. Note that $\omega_{b}$ is known and R(z) and V(z) contain odd-harmonics components. If the reference R(z) and disturbance V(z) have different basis frequencies, then multi period-based RCs such as in [29,30] can be utilized.*

The following remarks are also provided to describe the conservation of Assumptions 1 to 3:

**Remark 2.**  *The zero-phase low-pass filter q(z) is a moving average filter with a non-causal form. However, the presence of multiplication with a delay term (i.e, $z^{-N}$ or $z^{-N/2}$) makes the filter realizable (see Figure 1 and Figure 2).*

**Remark 3.**  *Assumption 2 reflects a model-based control system design. In this control system design, we require an open-loop plant model P(z).*

**Remark 4.**  *The exact values of the periodicity and the basis frequency of the reference R(z) and disturbance V(z) are required in the design. One may observe them using measurement instruments, including an oscilloscope and spectrum analyzer.*

Based on Assumptions 1 to 3, we have the information about q(z), $P_{s}\left(z\right)$ and $\omega_{b}$, which is needed for designing the proposed stabilizing controller F(z). Instead of using an inverse plant model [21,22,23], which is often not available due to plant uncertainties, the stabilizing controller F(z) can be designed in simple form as follows:

(14) $$\begin{array}{c} F\left(z\right)=K_{p}z^{M}, \end{array}$$

where $K_{p}$ is a positive learning gain and *M* is a positive number, which is not necessarily an integer. This means that *M* is possibly a fractional positive number. This is different to the references [24,28], where *M* is strictly an integer number. Here, we aim to obtain the stabilizing controller (14), satisfying the condition (13) given that q(z), $P_{s}\left(z\right)$ and $\omega_{b}$ are provided. The stability condition (13) is equivalent to

(15) $$\begin{array}{c} |\left\{1-F\left(z\right)P_{s}\left(z\right)\right\}q\left(z\right)|<1\;\forall z=e^{j\omega },\;0<\omega <\frac{\pi }{T}, \end{array}$$

where |X(z)| operates as the magnitude response of the transfer function X(z).

Let $N_{p}\left(\omega \right)$ and $\theta_{p}\left(\omega \right)$ be the magnitude and phase responses of $P_{s}\left(z\right)$. The transfer function $P_{s}\left(z\right)$ can be written as $P_{s}\left(z\right)=N_{p}\left(\omega \right)e^{j\theta_{p}\left(\omega \right)}$, while F(z) can be expressed as $F\left(z\right)=K_{p}e^{jM\omega }$. Following Assumption 1 that q(z) contributes a zero-phase for all frequencies, condition (15) can be rewritten as

(16) $$\begin{array}{c} |\left\{1-K_{p}e^{jM\omega }N_{p}\left(\omega \right)e^{j\theta_{p}\left(\omega \right)}\right\}N_{q}\left(\omega \right)|<1. \end{array}$$

Then, (16) can be further adjusted to

(17) $$\begin{array}{cc} |1-K_{p}N_{p}\left(\omega \right)e^{j\left[\theta_{p}\left(\omega \right)+M\omega \right]}|N_{q}\left(\omega \right) & <1\\ N_{q}\left(\omega \right)\sqrt{\left(1-K_{p}N_{p}\left(\omega \right)e^{j\left[\theta_{p}\left(\omega \right)+M\omega \right]}\right)\left(1-K_{p}N_{p}\left(\omega \right)e^{-j\left[\theta_{p}\left(\omega \right)+M\omega \right]}\right)} & <1\\ N_{q}\left(\omega \right)\sqrt{1-2K_{p}N_{p}\left(\omega \right)\cos\left[\theta_{p}\left(\omega \right)+M\omega \right]+{\left\{K_{p}N_{p}\left(\omega \right)\right\}}^{2}} & <1 \end{array}$$

Squaring both sides of (17), (17) becomes

(18) $$\begin{array}{c} N_{q}^{2}\left(\omega \right)\left(1-2K_{p}N_{p}\left(\omega \right)\cos\left[\theta_{p}\left(\omega \right)+M\omega \right]+{\left\{K_{p}N_{p}\left(\omega \right)\right\}}^{2}\right)<1. \end{array}$$

**Remark 5.**  *From (18), it can be seen that the ideal condition is achieved when $K_{p}N_{p}\left(\omega \right)\to 1$ and $\left[\theta_{p}\left(\omega \right)+M\omega \right]\to 0^{o}$ for $0<\omega <\frac{\pi }{T}$. This means that the magnitude compensation results in a unity gain and the phase compensation gives a zero phase. However, this condition is hard to achieve, especially at higher frequencies. The presence of q(z) offering $N_{q}\left(\omega \right)<<1$ makes it possible to satisfy condition (18).*

Now, we define $F(\omega_{j})$ as

(19) $$\begin{array}{c} F\left(\omega_{j}\right)=N_{q}^{2}\left(\omega_{j}\right)\left(1-2K_{p}N_{p}\left(\omega_{j}\right)\cos\left[\theta_{p}\left(\omega_{j}\right)+M\omega_{j}\right]+{\left\{K_{p}N_{p}\left(\omega_{j}\right)\right\}}^{2}\right), \end{array}$$

which represents a cost function in the left hand side of (18) assessed at a single frequency $\omega_{j}$. Since the repetitive model represents the reference/disturbance model at odd-harmonics only, the following objective function is constructed:

(20) $$\begin{array}{c} F_{T}=\sum_{j=0}^{L}F\left(\omega_{j}\right)\;\forall \;\omega_{j}=\left(2j+1\right)\omega_{b},\;L=\mathrm{ceil}\left((N-1)/4\right), \end{array}$$

where $\omega_{b}$ is the basis frequency given by Assumption 3 and *L* is an integer number calculated such that $\left(2L+1\right)\omega_{b}\approx \pi /T$. Finally, we present the optimization problem as follows:

(21) $$\begin{array}{cc} \; & \min_{K_{p},M}\begin{array}{cc} \; & F_{T}=\sum_{j=0}^{L}F\left(\omega_{j}\right) \end{array}\\ \; & \mathrm{subject}\;\mathrm{to}\\ \; & \left(1\right).\;\left(\begin{array}{c} 0\\ 0 \end{array}\right)<\left(\begin{array}{c} K_{p}\\ M \end{array}\right)\\ \; & \left(2\right).\;F\left(\omega_{j}\right)<1\;\forall \;\omega_{j}=\left(2j+1\right)\omega_{b},\;j=0,1,\cdots ,L. \end{array}$$

**Remark 6.**  *The optimization problem (21) aims to find two unknown variables $K_{p}$, M, which minimize the objective function (20) and satisfy two constraints. The first constraint is added to guarantee that the obtained $K_{p}$ and M are positive numbers. The second constraint is needed to ensure that the stability condition at each frequency $\omega_{j}$ is satisfied. Note that M is not necessarily an integer.*

### 3.4. Realization of the Controller

It is possible that the solution for variable *M* is a fractional number. Hence, the stabilizing controller F(z) is not realizable. To deal with this problem, the stabilizing controller F(z) is then split into:

(22) $$\begin{array}{c} F\left(z\right)=z^{M_{i}}\left[K_{p}z^{M_{f}}\right], \end{array}$$

where $M_{i}+M_{f}=M$, $M_{i}$ is positive integer number and $M_{f}$ is a negative fractional number, i.e., $M_{i}>M$ and $M_{f}<0$. The term $z^{M_{f}}$ can be approximated by a stable and causal IIR filter I(z) as follows:

(23) $$\begin{array}{c} z^{M_{f}}\approx I\left(z\right)=\frac{a_{R}z^{R}+a_{R-1}z^{R-1}+\cdots +a_{0}}{a_{0}z^{R}+a_{1}z^{R-1}+\cdots +a_{R}} \end{array}$$

where $R=\mathrm{ceil}(M_{f})$. The coefficients $a_{0},a_{1},\cdots ,a_{R}$ are designed by using Thiran fractional-delay formula [31,32]. The Thiran formula is given by

(24) a0=1,ak=−1jRk∏i=0RMf−R+iMf−R+k+i,∀k∈{1,2,⋯,R},

where

(25) Rk=R!k!R−k!.

If we pick $M_{i}=\mathrm{ceil}\left(M\right)$, then $M_{f}$ is within $-1<M_{f}<0$. Now, $z^{M_{f}}$ can be approximated as a first-order filter as follows:

(26) $$\begin{array}{c} R=1\to z^{M_{f}}\approx I\left(z\right)=\frac{a_{1}z+a_{0}}{a_{0}z+a_{1}z}. \end{array}$$

Here,

(27) $$\begin{array}{c} a_{0}=1,\;a_{1}=\frac{1-M_{f}}{M_{f}+1}. \end{array}$$

**Figure 5:**
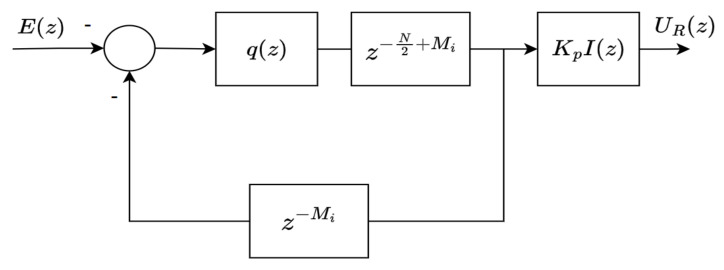
Realization of the proposed controller.

By applying (27), the IIR filter coefficients can be easily obtained. Since $z^{M_{i}}$ in (22) is non-causal, it cannot be implemented separately without being merged with the internal model. The modification shown in Figure 5 is presented to make the proposed controller fully realizable.

## 4. Simulation Results

The plant model of Quanser SRV02 servo in [33] is used in the simulation. The open-loop plant has the following model:

(28) $$\begin{array}{c} P\left(s\right)=\frac{\theta_{o}\left(s\right)}{V_{o}\left(s\right)}=\frac{1.74}{s\left(0.0268s+1\right)}\;, \end{array}$$

where $\theta_{o}\left(s\right)$ is an angle position (rad) and $V_{o}\left(s\right)$ is an open-loop voltage (V). The plant model (28) is sampled with zero-order-hold method at the sampling period T=0.005s, resulting in the discrete-time model as follows:

(29) $$\begin{array}{c} P\left(z\right)=10^{-4}\frac{7.634z+7.7173}{\left(z-1)(z-0.8288\right)}\;, \end{array}$$

which is a marginally stable plant since P(z) has one of its poles lying on the unit circle. Recalling (10), we add a simple proportional gain D(z)=10 to improve the stability margin of (29). As a result, it leads to the minimal realization of the closed-loop plant model $P_{s}\left(s\right)$ given as

(30) $$\begin{array}{c} P_{s}\left(z\right)=10^{-3}\frac{7.634z+7.7173}{z^{2}-1.822z+0.837}\;. \end{array}$$

**Figure 6:**
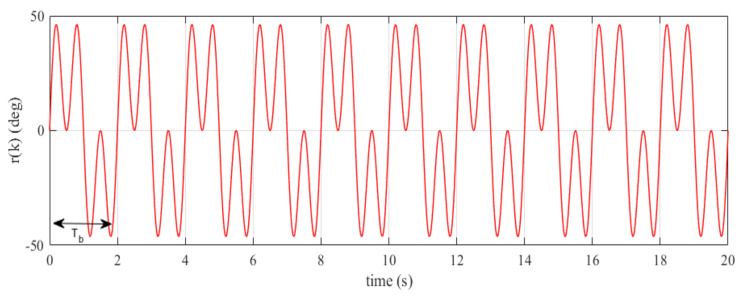
Reference signal r(k).

Now, we have a second-order discrete-time model as shown in (30). The closed-loop plant model $P_{s}\left(z\right)$ has two stable complex poles located at $p_{1}=0.91+i0.084$ and $p_{2}=0.91-i0.084$. Hence, Condition 2 (C2) in Remark 1 is satisfied. Here, we consider a tracking control problem with no presence of disturbance (i.e., V(z)=0). A periodic reference signal r(k) with a maximum amplitude 0.81 rad (46.19 deg) as illustrated in Figure 6 is defined as a reference input of the system expressed as

(31) $$\begin{array}{c} r\left(k\right)=\frac{\pi }{6}\sin\left(\pi kT_{s}\right)+\frac{\pi }{6}\sin\left(3\pi kT_{s}\right) \end{array}$$

It can be seen from (31) that the basis frequency of r(k) is $\omega_{b}=\pi$ rad/s. Consequently, the integer number *N* is computed as $(2\pi /\omega_{b}T_{s})=400$. The q-filter q(z) is selected as $q\left(z\right)=0.25z^{-1}+0.5+0.25z$, which is a stable low-pass filter with a cut-off frequency as $\omega_{q}=228$ rad/s. Using $P_{s}\left(z\right)$, *N* and q(z), the optimization problem (21) can now be constructed. Utilizing the Optimization Toolbox by MATLAB, the optimization problem can be solved resulting in the stabilizing controller as follows:

(32) $$\begin{array}{c} F\left(z\right)=1.131z^{7.927} \end{array}$$

Following the steps (22) to (27), we get

(33) $$\begin{array}{c} M_{i}=8,\;M_{f}=-0.073,\;z^{M_{f}}\approx I\left(z\right)=\frac{0.864z+1}{z+0.864} \end{array}$$

Here, the IIR filter I(z) has a single pole at $p_{1}=-0.864$, confirming that $z^{M_{f}}$ is replaced by a stable and causal filter. Hence, F(z) in (32) can now be approximated by

(34) $$\begin{array}{c} F\left(z\right)\approx z^{8}\left[1.131\frac{0.864z+1}{z+0.864}\right]. \end{array}$$

Finally, the transfer function of the proposed controller can be represented as

(35) $$\begin{array}{c} C_{o}\left(s\right)=-\frac{\left(0.25z^{-1}+0.5+0.25z\right)z^{-192}}{1+\left(0.25z^{-1}+0.5+0.25z\right)z^{-200}}\left[1.131\frac{0.864z+1}{z+0.864}\right]. \end{array}$$

**Figure 7:**
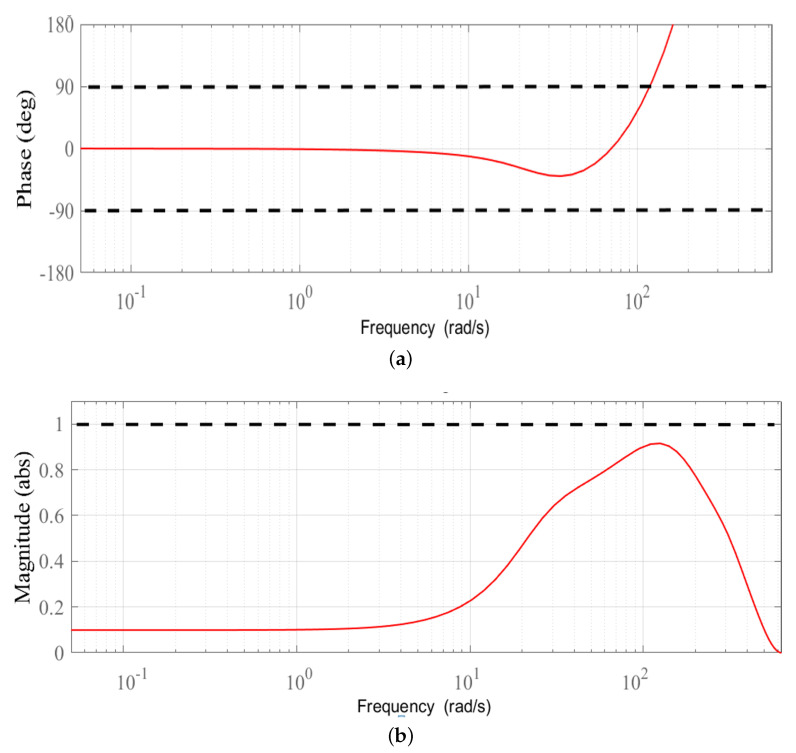
(**a**) Phase response of $F\left(z\right)P_{s}\left(z\right)$ (**b**) magnitude response of $\{1-F\left(z\right)P_{s}\left(z\right)\}q\left(z\right)$.

**Figure 8:**
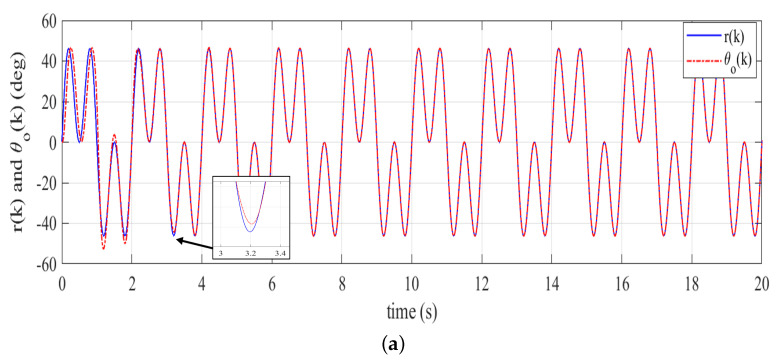

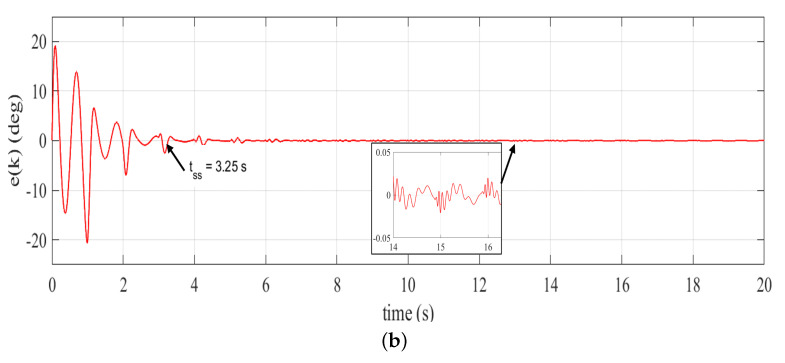
Tracking performance of the proposed controller (simulation), (**a**) reference and tracking output, (**b**) tracking error.

Based on Remark 2, we firstly examine the phase compensation and the stability condition provided by the stabilizing controller F(z) given in (34). The phase compensation and the stability condition are plotted in Figure 7a,b, respectively. As shown in Figure 7a, the proposed stabilizing controller F(z) provides phase compensation with wider stable range $\left[-90^{\circ },90^{\circ }\right]$ to approximately 200 rad/s. The overall system is guaranteed to be stable due to the magnitude responses of $\{1-F\left(z\right)P_{s}\left(z\right)\}q\left(z\right)$ being less than one for all frequencies. This behavior is clearly indicated from Figure 7b. The tracking output and tracking error plots, respectively, given in Figure 8a,b demonstrate that the reference signal r(k) with odd-harmonics frequencies is accurately tracked after about 3.25 s. Figure 8b also indicates a significantly small steady-state tracking error; that is, $|e\left(k\right)|<0.05^{\circ }$. These simulation results show that the proposed method effectively works for tracking/rejection of odd-harmonics repetitive control system.

## 5. Experimental Validation

**Figure 9:**
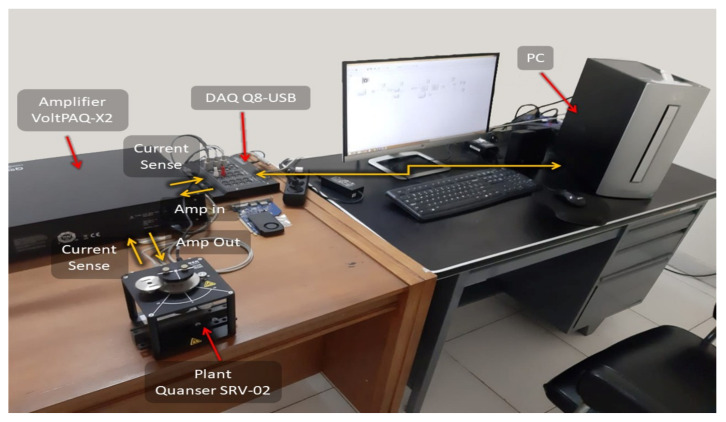
Experimental Setup.

The real-time experiments are conducted to further validate the effectiveness of the proposed design. Figure 9 shows an experimental setup consisting of PC (as host PC and target system), DAQ Q8-USB (as ADC and DAC), Amplifier VoltPAQ-X2 (as two channels signal conditioning) and Quanser SRV-02 (as an open-loop servomotor plant). The open-loop plant model is similar to the model (28) used in the simulation. In the experiments, we aim to regulate the servomotor SRV-02 such that the angle position $\theta_{o}\left(k\right)$ (deg) accurately follows the periodic reference r(k) consisting of odd-harmonics frequencies as shown in Figure 6.

In the experiments, we compare the performance of the proposed controller with general RC using a similar stabilizing controller. The compared general RC has the following transfer function:

(36) $$\begin{array}{c} C_{g}\left(z\right)=\frac{\left(0.25z^{-1}+0.5+0.25z\right)z^{-400}}{1-\left(0.25z^{-1}+0.5+0.25z\right)z^{-400}}F\left(z\right). \end{array}$$

**Figure 10:**
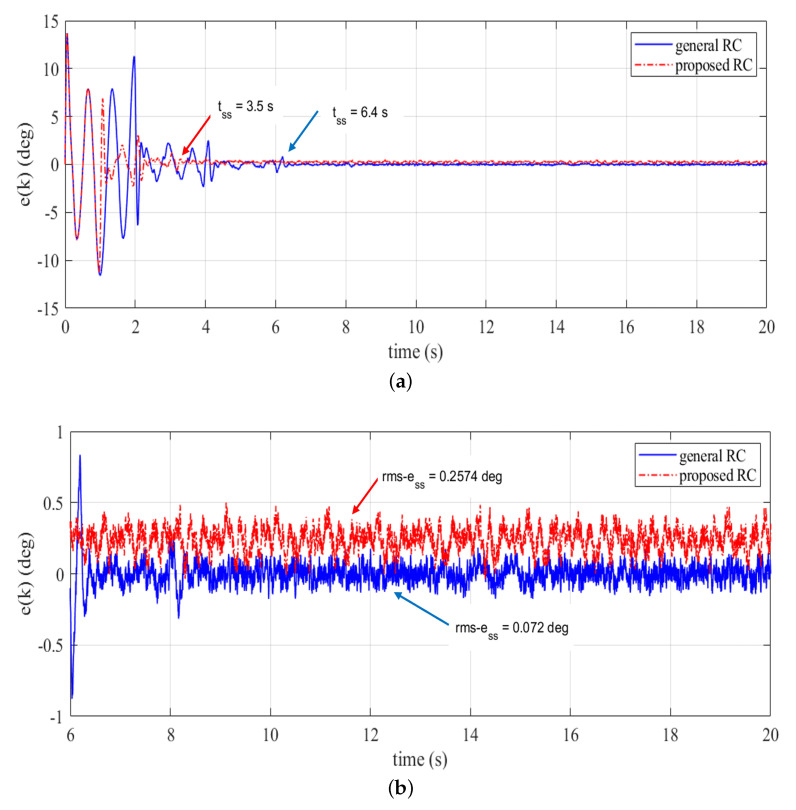
Tracking errors (experimental validation), (**a**) transient + steady-state, (**b**) steady-state.

Here, F(z) in (36) is given as in (34). In addition, we also examine the root-mean-square error (rmse) and the root-mean-square of steady-state error (rms-ess) defined as:

(37) $$\begin{array}{cc} \mathrm{rmse} & :=\sqrt{\frac{1}{n_{T}}\sum_{k=1}^{n_{T}}e{\left(k\right)}^{2}}, \end{array}$$

(38) $$\begin{array}{cc} \mathrm{rms}-\mathrm{ess} & :=\sqrt{\frac{1}{\left(n_{T}-n_{ss}\right)}\sum_{k=n_{ss}}^{n_{T}}e{\left(k\right)}^{2}}, \end{array}$$

where $n_{T}=20/0.005=4000$, $n_{ss}=t_{ss}/0.005$ and $t_{ss}$ is time to reach the steady-state condition. The tracking errors of the proposed RC and general RC from the experimental validation are plotted in Figure 10. The error plots given in Figure 10a show that the proposed RC has a superior transient response compared to the general RC. The proposed RC converges after about 3.5 s, which is almost half of the convergence time of the general RC (i.e., 6.4 s). This phenomenon is obvious because the delay period of general RC (36) is twice that of the proposed controller in (35). However, the general RC provides better tracking accuracy compared to the proposed RC. This is noticeable from the steady-state error shown in Figure 10b. We can also notice that the calculated rms-ess value of the general RC is smaller compared to that of the proposed RC.

We also evaluate the performance of both controllers when some integer phase lead stabilizing controllers are considered. It can be seen from Table 1, that the proposed controller gives the smallest rmse value, showing the superiority over the general RC and OHRC with integer phase lead stabilizing controller.

Another comparison is also made to the integer phase lead RC developed in [24]. The stabilizing controller in [24] has the following form:

(39) $$\begin{array}{c} F_{p}\left(z\right)=k_{p}z^{m}, \end{array}$$

where $k_{p}$ is a learning gain and *m* is an integer lead step. In [24], the design task is started by choosing the lead step *m* which gives larger stable bandwidth and satisfies the following phase condition:

(40) $$\begin{array}{c} |\theta_{p}\left(\omega \right)+m\omega |<90^{o} \end{array}$$

**Figure 11:**
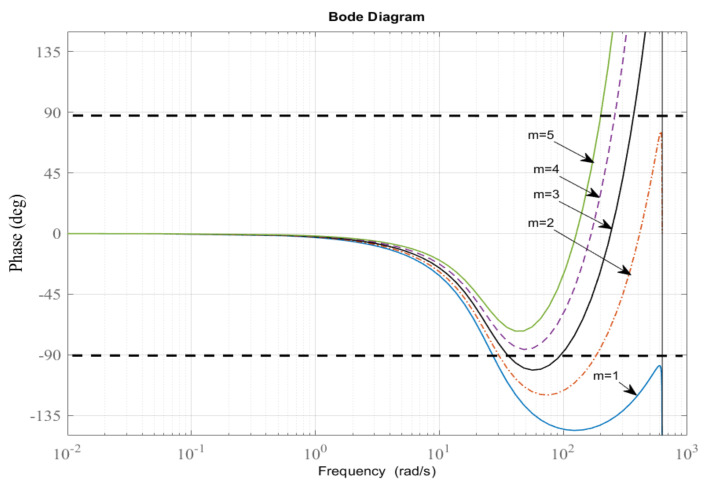
Phase responses of $z^{m}P_{s}\left(z\right)$.

Here, the lead step *m* is assessed one by one, by plotting the phase responses shown in Figure 11. As depicted in Figure 11, the lead step m=4 is chosen. Then, the learning gain $k_{p}$ is manually tuned to give fast convergence and theoretically determined to meet the magnitude condition as follows:

(41) $$\begin{array}{c} k_{p}<\frac{2\cos(\theta_{p}\left(\omega \right)+m\omega )}{N_{p}\left(\omega \right)}, \end{array}$$

where *m* is the chosen lead step (i.e., m=4), and $N_{p}\left(\omega \right)$ and $\theta_{p}\left(\omega \right)$ are, respectively, the magnitude response and the phase response of the closed-loop plant $P_{s}\left(z\right)$.

**Figure 12:**
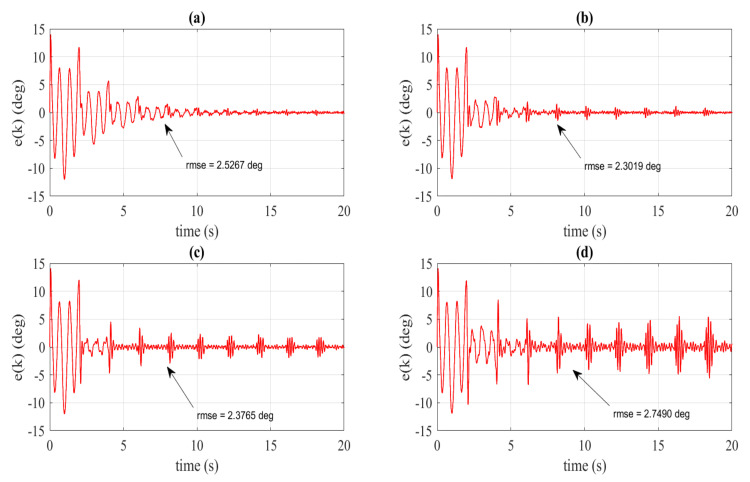
Tracking errors (experimental validation) of phase lead RC $k_{r}z^{4}$ for different learning gains (**a**) $k_{p}=0.5$, (**b**) $k_{p}=0.75$, (**c**) $k_{p}=1$, (**d**) $k_{p}=1.25$.

**Figure 13:**
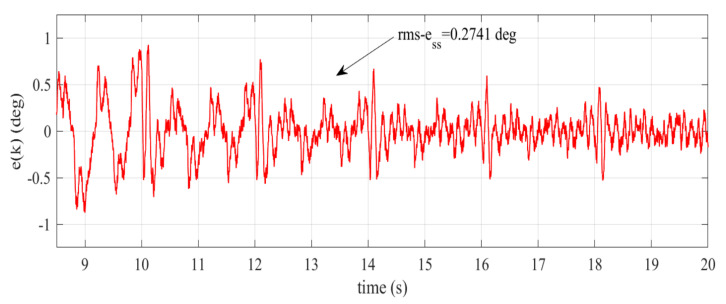
Steady-state error of the phase-lead RC at the gain $k_{p}=0.5$.

Let us manually pick the learning gains $k_{p}$ as 0.5,0.75, 1 and 1.25. The tracking errors of the integer phase-lead RC system [24] with different learning gains are shown in Figure 12a–d. Figure 12a–d indicate that even though the learning gain is increased, the phase lead RC system remains, showing slower convergence rate compared to the proposed RC. In addition, the calculated root-mean-square errors of the phase lead RC system with different learning gains are significantly larger compared to the proposed RC. We can also notice that increasing the learning gain results in larger steady-state error. As observed from Figure 12d, that tracking error tends to diverge after t=10s. This implies that increasing the learning gain to 1.25 leads to an unstable closed-loop system. The steady-state error and the rms-ess value of the phase-lead RC, especially at the gain $k_{p}=0.5$, are also examined. Figure 13 indicates that the phase-lead RC has a bigger range of steady-state error. Moreover, the rms-ess of phase-lead RC is also larger than in the proposed RC. All the above results demonstrate the superiority of the proposed controller over the general RC (3) and the integer phase-lead RC [24].

## 6. Conclusions

In this paper, a discrete-time fractional-order odd-harmonics repetitive controller has been developed. First, an internal model with a half-cycle delay is used to track/compensate periodic signals with odd-harmonics components. Second, the fractional-order phase lead stabilizing controller is designed based on the optimization problem derived from the RC’s stability condition. Finally, the fractional term of the stabilizing controller is approximated by a causal and stable IIR filter, with coefficients that are calculated by using the Thiran formula. Simulation, experimental validation and comparison study were conducted to verify the effectiveness of the proposed design. 

## Figures and Tables

**Table 1:** Root-mean-square error (rmse) analysis (hardware experiments).

Stabilizing Controller	rmse (deg)-General RC	rmse (deg)-OHRC
$1.131z^{7.927}$	2.189	1.599
$1.131z^{8}$	2.207	1.605
$1.131z^{7}$	2.224	1.616
$1.131z^{6}$	2.247	1.656

## Data Availability

Not applicable.
